# Thickness Considerations of Two-Dimensional Layered Semiconductors for Transistor Applications

**DOI:** 10.1038/srep29615

**Published:** 2016-07-12

**Authors:** Youwei Zhang, Hui Li, Haomin Wang, Hong Xie, Ran Liu, Shi-Li Zhang, Zhi-Jun Qiu

**Affiliations:** 1 State Key Laboratory of ASIC and System, School of Information Science and Technology, Fudan University, Shanghai 200433, China; 2 State Key Laboratory of Functional Materials for Informatics, Shanghai Institute of Microsystem & Information Technology, Chinese Academy of Sciences, Changning Road 865, Shanghai 200050, China; 3 Solid-State Electronics, The Ångström Laboratory, Uppsala University, Uppsala Box 534, SE-751 21, Sweden

## Abstract

Layered two-dimensional semiconductors have attracted tremendous attention owing to their demonstrated excellent transistor switching characteristics with a large ratio of on-state to off-state current, *I*_on_/*I*_off_. However, the depletion-mode nature of the transistors sets a limit on the thickness of the layered semiconductor films primarily determined by a given *I*_on_/*I*_off_ as an acceptable specification. Identifying the optimum thickness range is of significance for material synthesis and device fabrication. Here, we systematically investigate the thickness-dependent switching behavior of transistors with a wide thickness range of multilayer-MoS_2_ films. A difference in *I*_on_/*I*_off_ by several orders of magnitude is observed when the film thickness, *t*, approaches a critical depletion width. The decrease in *I*_on_/*I*_off_ is exponential for *t* between 20 nm and 100 nm, by a factor of 10 for each additional 10 nm. For *t* larger than 100 nm, *I*_on_/*I*_off_ approaches unity. Simulation using technical computer-aided tools established for silicon technology faithfully reproduces the experimentally determined scaling behavior of *I*_on_/*I*_off_ with *t*. This excellent agreement confirms that multilayer-MoS_2_ films can be approximated as a homogeneous semiconductor with high surface conductivity that tends to deteriorate *I*_on_/*I*_off_. Our findings are helpful in guiding material synthesis and designing advanced field-effect transistors based on the layered semiconductors.

The first successful demonstration of field-effect transistors (FETs) based on monolayer molybdenum disulfide (MoS_2_) with appealing performance^1^,^2^ has stimulated intensive research on two-dimensional (2D) transition metal dichalcogenides (TMDs). The planar nature of these 2D semiconductor materials could potentially lead complementary metal-oxide-semiconductor (CMOS) technology to the ultimate size scaling envisioned by Moore’s law and beyond^3^,^4^,^5^. MoS_2_, a representative layered TMD, has a satisfactory bandgap in the range of 1.3 to 1.8 eV^6^,^7^, which is advantageous over the well-studied gapless graphene with respect to the standby leakage current of its FETs^8^. The bandgap of MoS_2_ is thickness-dependent and it is 1.8 eV for monolayers. As a result, transistors of both single- and multilayer-MoS_2_ films have an exhibited high ratio of on-state to off-state current (*I*_on_/*I*_off_ > 10^6^) with reasonable electron mobility^1^,^9^,^10^,^11^. All this makes the layered TMDs promising in fields of low-power switches/circuits^11^,^12^, nonvolatile memory devices^13^,^14^, ultrasensitive photodetectors^15^,^16^, *etc*.

In FET applications, multilayer MoS_2_ with a smaller bandgap is of greater potential than the monolayer counterpart^17^. First, multilayer MoS_2_ has a 3-fold higher density of states and conducts current along multiple channels, which can be translated to a considerably high drive current^11^,^18^. Second, the interlayer screening effect leads to a higher carrier mobility^19^,^20^ and better noise immunity^21^ in multilayer MoS_2_. Compared to the direct-bandgap monolayer MoS_2_ requiring a strict thickness control, the electronic properties of multilayer MoS_2_ manifested by an indirect bandgap are relatively insensitive to layer thickness. Hence, multilayer MoS_2_ is better suited for large-area and/or high-density electronics^22^,^23^,^24^. However, multilayer MoS_2_ and other TMD semiconductors have so far gained limited attention for their use in electronics, compared to their monolayer counterparts.

Usually, MoS_2_ is an *n*-type semiconductor that is determined by both intrinsic and extrinsic effects, such as sulfur vacancies, impurities or other structural defects^25^,^26^,^27^. Control of the electron mobility and carrier density in MoS_2_ FETs can be achieved by engineering on the gate structure and dielectrics^28^,^29^,^30^. In multilayer MoS_2_, the gate control of the electron density in the channel is weakened with increasing MoS_2_ thickness. When the MoS_2_ thickness is larger than the maximum depletion width, *W*_max_, the electrostatic gating loses control over the electrons in the excess part of the MoS_2_ film beyond *W*_max_. This situation is well known in the partially depleted silicon-on-insulator (SOI) technology. If there is no energy barrier at the source or drain terminals, the electrons in this excess MoS_2_ will contribute significantly to the leakage current. The potential advantages of multilayer MoS_2_ are, then, offset by a high *I*_off_, leading to a significantly reduced *I*_on_/*I*_off_. Hence, identifying the optimum thickness range is of significance for material synthesis and practical device application of the 2D TMDs. For digital logic applications, *I*_on_/*I*_off_ of at least 10^3^ is generally required^31^. In this work, we will use this *I*_on_/*I*_off_ = 10^3^ ratio as a design criterion, especially when an optimum layer thickness is concerned for achieving high performance with acceptable *I*_on_/*I*_off_. In view of performance variation associated with probable non-uniformity with unintentional *n*-doping in the starting MoS_2_ material as well as from device fabrication, this work builds on a statistical study of the key device parameters in order to gain a comprehensive understanding of the switching properties. Specifically, we investigate the thickness-dependent *I*_on_/*I*_off_ in multilayer MoS_2_ by a statistical analysis of more than 80 devices in a wide thickness range. Differences in *I*_on_/*I*_off_ are large amounting to several orders of magnitude with increasing layer thickness. The optimum layer thickness is defined by *W*_max_, beyond which *I*_on_/*I*_off_ is reduced below 10^3^.

## Results

**Figure 1:**
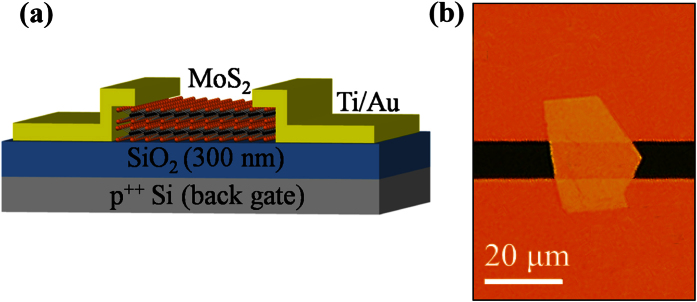
(**a**) Schematic representation and (**b)** Optical image of the field-effect transistor based on multilayer MoS_2_.

**Figure 2:**
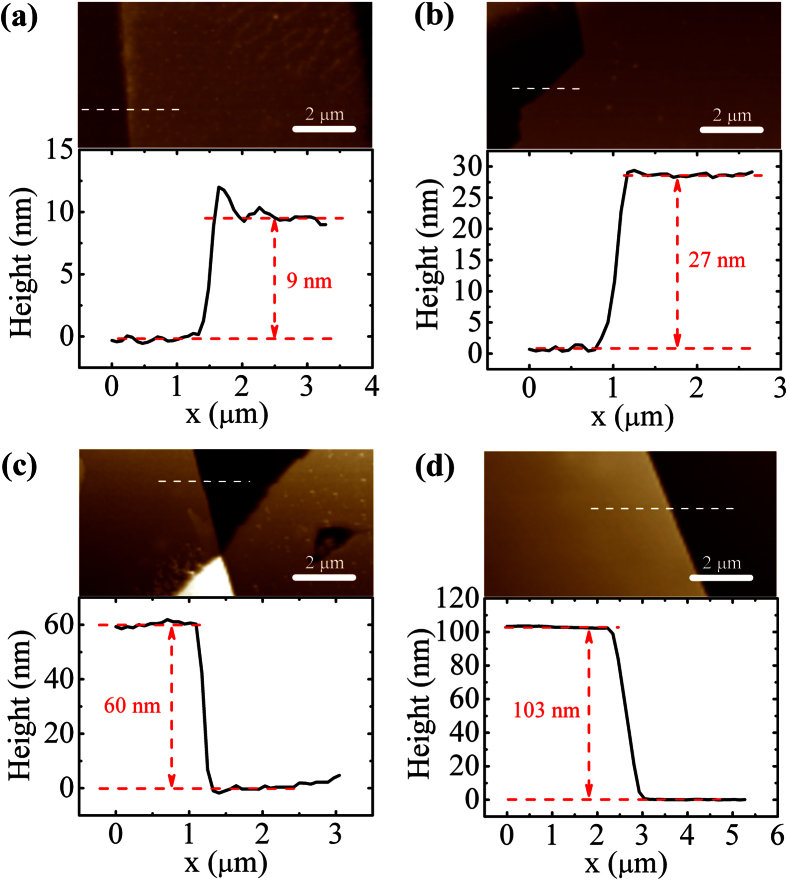
(**a–d**) AFM images of 9, 27, 60 and 103-nm thick MoS_2_ films on SiO_2_/Si substrate. The height profiles are measured along the dashed lines in the images.

A schematic representation of a back-gate multilayer-MoS_2_ transistor used in our work is shown in Fig. 1a, whereas a typical top view photomicrograph of a fabricated device is given in Fig. 1b. Isolated MoS_2_ flakes on the SiO_2_/Si substrate were exfoliated from a bulk MoS_2_ crystal using a conventional mechanical exfoliation technique^32^. The sample preparation and device fabrication are detailed in Methods. The thickness of different flakes, *t*, was measured by means of atomic force microscopy (AFM), as illustrated in Fig. 2. Only one channel length of 10 μm is used for all devices and it is defined by the spacing of a Cu grid shadow mask. However, the channel width that is determined by the width of the flakes varies in the range of 2–60 μm as a result of the stochastic nature of the MoS_2_ exfoliation process. The source and drain metal used in our devices is 50 nm Au with a 5 nm thick Ti adhesion layer. The Ti adhesion layer that is in intimate contact with MoS_2_ has a work function of ∼4.3 eV, which is very close to the conduction band edge of thin-layer MoS_2_^9^,^33^. Furthermore, Ti is a transition metal with its *d*-electron orbitals mixing favorably with the 4*d* states of Mo and resulting in an increase in the density of states at the Fermi level and a strong Fermi level pinning at the contact^9^,^34^,^35^. Therefore, this favorable interface geometry is expected to facilitate a good chemical bonding and allow for a maximized electron injection at the source/drain contacts with an increased overlap between the states at the interface.

**Figure 3:**
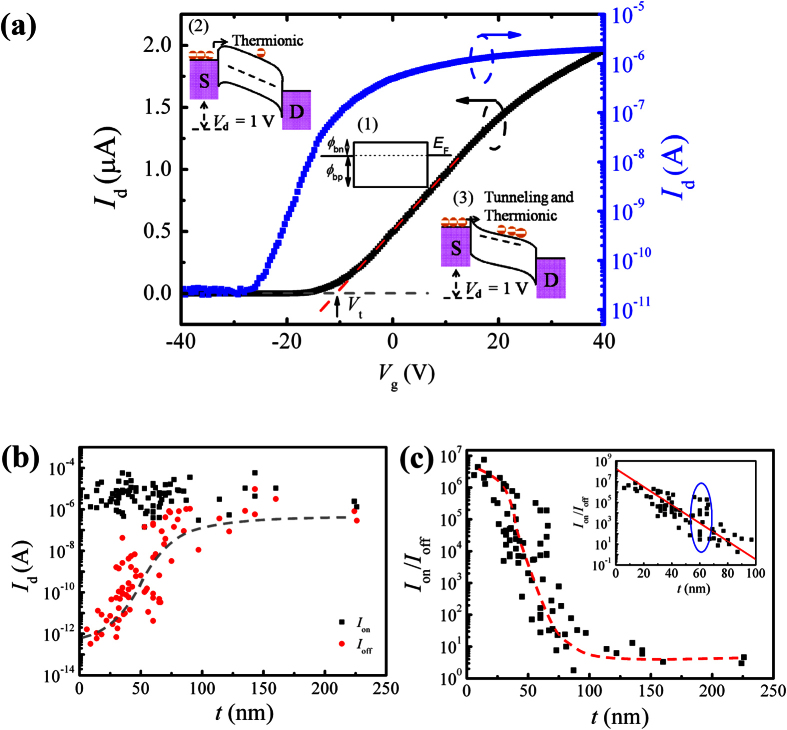
(**a**) Transfer characteristics of a representative transistor with a 30-nm-thick MoS_2_ film on a linear scale (left y-axis) and a log scale (right y-axis). The threshold voltage, *V*_t_, is determined by the intercept on the x-axis with the regression fitted line to the linear scale characteristics. The insets represent the energy band diagrams corresponding to the applied *V*_g_ in three distinct regions: (1) at flat band, (2) below threshold, and (3) above threshold. *ϕ*_bn_ and *ϕ*_bp_ indicate electron and hole SBH, respectively. (**b**) Dependence of *I*_on_ and *I*_off_ on MoS_2_ flake thickness. The grey dashed line serves as a guide to the eye. (**c**) Thickness-dependence of *I*_on_/*I*_off_. The red dashed line serves as a guide to the eye. Inset is the zoom in for the first 100 nm. The red solid line in the inset is a linear fit on the semi-log scale.

The transfer characteristics of a multilayer-MoS_2_ FET shows a typical *n*-type unipolar carrier transport behavior (Fig. 3a). This confirms a rather small (<0.1 eV), if not negligible, Schottky barrier height (SBH) for electrons at the Au/Ti-MoS_2_ contacts (Supplementary Fig. S1). The SBH for holes is, thus, high (>1.2 eV) since the sum of electron SBH and hole SBH should approximately be equal to the energy bandgap (1.3 eV) of MoS_2_ (inset in Fig. 3a). Tunneling through the Schottky barrier at the metal/MoS_2_ contacts not only limits the charge injection in the device at its on-state but also plays a critical role when evaluating the device off-state in the subthreshold region of the transistor. The small electron SBH facilitates the injection of accumulated electrons at positive gate voltage, *V*_g_, while the large hole SBH suppresses the injection of inverted holes at negative *V*_g_. This results in the *n*-type unipolar behavior with a high *I*_on_/*I*_off_ and is in stark contrast to the ambipolar conduction behavior in graphene FETs with a low *I*_on_/*I*_off_^32^. The FET with a 30-nm-thick multilayer MoS_2_ in Fig. 3a operates as a depletion-mode FET with a large negative threshold voltage, *V*_t_, and a high *I*_on_/*I*_off_ of 10^5^. All the FETs fabricated in this work exhibit the same *n*-type characteristics, regardless of the thickness of the MoS_2_ film in channel (Supplementary Fig. S2).

The depletion-mode nature of the transistors will set a limit on the thickness of the multilayer MoS_2_ films primarily determined by the preset *I*_on_/*I*_off_ = 10^3^ as an acceptable specification. Over 80 multilayer-MoS_2_ FETs were fabricated and characterized. Their *I*_on_ and *I*_off_ versus *t* ranging from 6 nm to 225 nm are plotted in Fig. 3b. The minimum *I*_off_ occurring for the smallest *t* is limited by the noise level in the devices. A clear trend is observed for *I*_off_; it increases rapidly with increasing *t* below 100 nm. Beyond *t* = 100 nm, *I*_off_ becomes comparable with *I*_on_ and is almost independent of layer thickness. The stochastic variation of the flake widths makes the variation of *I*_on_ with *t* unspecific. However, *I*_on_/*I*_off_ is unaffected by the width variation due to the same width-dependence of *I*_on_ and *I*_off_^36^. In the first 20–30 nm, *I*_on_/*I*_off_ exhibits a gradual decrease with increasing *t*, see Fig. 3c, likely caused by the *I*_off_ variation. This is followed by an exponential decrease in *I*_on_/*I*_off_ with *t* until it approaches unity for *t* > 100 nm. This is better seen in the inset of Fig. 3c where the best linear fit to the logarithmic *I*_on_/*I*_off_ versus *t* in the range of 20–100 nm gives


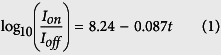


**Figure 4:**
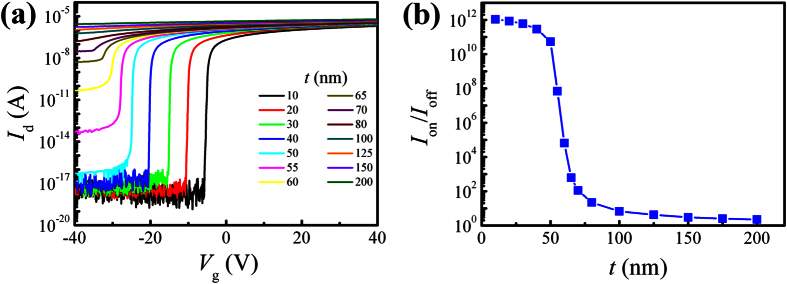
(**a**) Simulated transfer curves of FETs with various MoS_2_ thicknesses. (**b**) Simulated thickness-dependence of *I*_on_/*I*_off_.

It is well known that in the monolayer 2D materials, the charge carriers are confined in the 2D planes. This confinement can result in some unique characteristics not common in 3D materials, *e.g.* Si. When the layer thickness is increased, the carriers can hop freely between neighboring layers and move in the whole 2D layered material^37^. As a result, the carriers distribute fairly uniformly in the 2D material. In this aspect, multilayer-MoS_2_ films can be approximated as a homogeneous semiconductor and simulated with traditional device simulators. We have therefore used a commercial simulation tool SILVACO TCAD^38^ to numerically solve the coupled Poisson and continuity equations for the multilayer-MoS_2_ FETs. Our focus here is on charge and current distributions in MoS_2_. For simplicity, the electron SBH is set to 0.1 eV and the unintentional *n*-doping concentration, *N*_d_, in MoS_2_ is assumed to be 3.5 × 10^17^ cm^−3^, in order to attain identical *V*_t_ between the simulation and experiments. The doping concentration^39^ in MoS_2_ is found to vary from 10^16^ to 10^19^ cm^−3^. The other material parameters used in the simulation are shown in Supplementary Table S1^11^,^39^,^40^. The simulated transfer characteristics of multilayer-MoS_2_ FETs for various channel thicknesses (Fig. 4a) and the variation of *I*_on_/*I*_off_ with *t* (Fig. 4b) are in good agreement with the experimental results. In particular, the simulated *I*_on_/*I*_off_ shows a steep decrease with *t* around ~50 nm, matching very well with the data in Fig. 3c. This critical thickness is strongly correlated with *W*_max_ that is related to *N*_d_ by the following formula^36^:


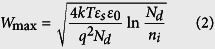


where, *k* is the Boltzmann constant, *T* is absolute temperature, *q* is elementary charge, *ε*_s_ is the relative dielectric constant of multilayer MoS_2_ (~11)^39^,^41^, *ε*_0_ is the vacuum permittivity, and *n*_i_ = 1.6 × 10^8^ cm^−3^ is the intrinsic carrier concentration in MoS_2_ due to thermal interband excitation^11^. The calculated *W*_max_ with the given *N*_d_ is ~60 nm. The spatial distribution of charge carriers is inhomogeneous, due to charge screening, along the depth of the multilayer-MoS_2_ film. Specifically, the charge carriers in the MoS_2_ layers close to the SiO_2_ interface are effectively controlled by *V*_g_. The electrostatic gate control of the carriers are weakened gradually, or even completely lost, in the MoS_2_ layers further away from the SiO_2_ interface on the account of charge screening.

**Figure 5:**
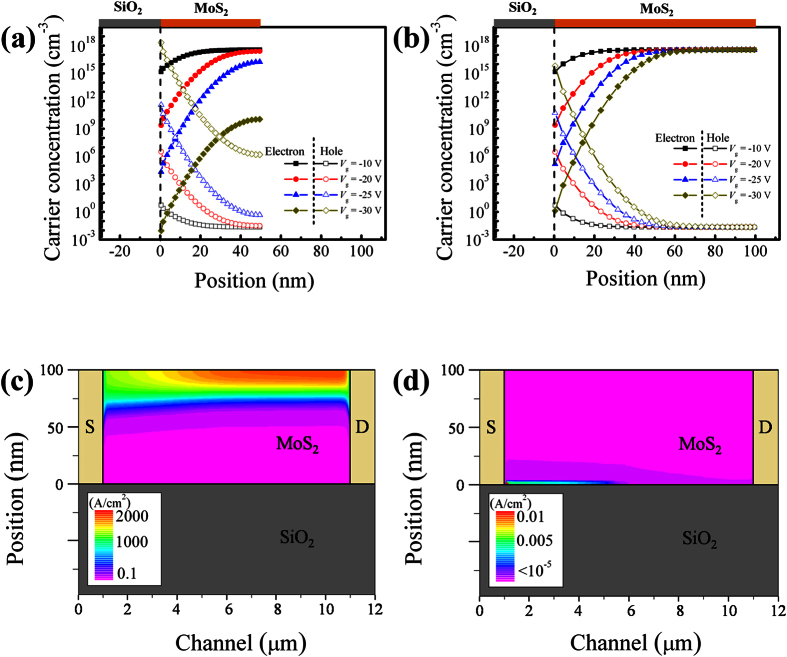
Carrier distribution in 50-nm (**a**) and 100-nm (**b**) MoS_2_ at various *V*_g_. (**c,d**) Electron and hole current distributions in a 100-nm-thick MoS_2_ at *V*_g_ = −30 V.

As the device switching behavior is mainly determined by *I*_off_, the carrier distribution at negative *V*_g_ is shown, respectively, in Fig. 5a,b for *t* < *W*_max_ and *t* > *W*_max_. At a negative *V*_g_, electrons are repelled from while holes are attracted to the MoS_2_/SiO_2_ interface. For *t* < *W*_max_, electrons are depleted in the whole MoS_2_ film at *V*_g_ < −25 V. Simultaneously, an inversion layer populated with holes is formed at the MoS_2_/SiO_2_ interface. However, when *t* > *W*_max_, the carrier concentration close to the sample surface (away from the MoS_2_/SiO_2_ interface) remains constant independent of *V*_g_. This is a result of charge screening effect that results in a poor electrostatic control of the electrons in the excess part of the MoS_2_ film. This high and uncontrolled electron concentration in this excess MoS_2_ close to the sample surface, shown in Fig. 5c, contributes to *I*_off_. Although a hole inversion layer is formed under large negative *V*_g_, the hole conduction current can be neglected due to a large SBH at the contact interfaces (see Fig. 5d).

It is established now that *W*_max_ is an important parameter determining the switching behavior of multilayer-MoS_2_ FETs. When *t* ≪ *W*_max_, the electrons can be fully depleted from the entire channel region under negative *V*_g_ and an excellent switching behavior with a very low leakage current prevails. Under such circumstances, *I*_on_/*I*_off_ is rather insensitive to *t*, as manifested by the slowly descending *I*_on_/*I*_off_ with *t* shown in Figs 3c and 4b. When *t* around *W*_max_, the electrons in the excess part of the MoS_2_ film cannot be fully depleted easily. An exponential decrease in *I*_on_/*I*_off_ by about 4 orders of magnitude with *t* changing from 30 to 65 nm. Moreover, *W*_max_ depends on doping concentration. The stochastic doping concentration in the MoS_2_ flakes can induce a variation in *W*_max_, which in its turn results in a large spread in the switching property at ~60 nm (indicated by a circle in the inset of Fig. 3c).

## Discussion

In view of its significance in device physics, design and fabrication, quantifying the transistor switching behavior as a function of the layer thickness of the 2D semiconductor materials is of vital importance. A critical parameter *W*_max_ is discussed when characterizing layered TMD semiconductors such as MoS_2_ as the channel material in FETs. Both experimental and theoretical studies show *W*_max_ = 50–60 nm for the multilayer-MoS_2_ FETs. At this thickness, the FETs are characterized by an acceptable *I*_on_/*I*_off_ around 10^3^. If the multilayer-MoS_2_ film is thinner than this *W*_max_, an excellent switching behavior with a low leakage current and a much higher *I*_on_/*I*_off_ (than 10^3^) will prevail. If the multilayer-MoS_2_ film is substantially thicker than this *W*_max_, a large leakage current will persist and the switching behavior becomes inferior. An exponential decrease in *I*_on_/*I*_off_ is found in the 20–100 nm thickness range, by a factor of 10 for each additional 10 nm. The findings in this work are useful in guiding material synthesis and designing advanced FETs with layered TMD semiconductors. It appears that the device physics established for traditional semiconductors such as Si is applicable for the layered TMD semiconductors, as it should be.

## Methods

Thin MoS_2_ flakes were peeled off from bulk MoS_2_ (SPI supplies) by mechanical exfoliation. They were subsequently transferred to a heavily doped *p*-type Si substrate with a 300-nm-thick thermally grown SiO_2_. The SiO_2_/Si substrate was pre-cleaned by sonication in acetone, isopropyl alcohol, and deionized water. The transferred MoS_2_ flakes were identified using an optical microscope (Keyence digital microscope VHX-600). The thickness of the MoS_2_ flakes was measured using AFM (Dimension 3100 with Nanoscope IIIa controller, Veeco) operated in tapping mode under ambient conditions. In order to avoid contamination from photolithography or electron-beam lithography, a 10-μm spacing copper grid was placed on top of the thin MoS_2_ flakes as a shadow mask for the electrode fabrication. A bilayer stack Ti/Au of 5/50 nm thickness was then deposited by means of electron-beam evaporation as the source and drain electrodes. The heavily doped Si substrate was used as the common back gate for the fabricated MoS_2_ FETs. Electrical characterization of the devices was carried out in a shielded probe station with Keithley 4200 semiconductor characterization system in ambient environments.

## Additional Information

**How to cite this article**: Zhang, Y. *et al.* Thickness Considerations of Two-Dimensional Layered Semiconductors for Transistor Applications. *Sci. Rep.* **6**, 29615; doi: 10.1038/srep29615 (2016).

## Supplementary Material

Supplementary Information
