# Supplementary material for: Thickness Considerations of Two-Dimensional Layered Semiconductors for Transistor Applications
Source: Sci Rep. 2016 Jul 12;6:29615. doi: 10.1038/srep29615 (PMC4940740; doi:10.1038/srep29615)
Supplement: Supplementary Information [file srep29615-s1.pdf]

Thickness Considerations of Two-Dimensional Layered Semiconductors for Transistor Applications

Youwei Zhang, Hui Li, Haomin Wang, Hong Xie, Ran Liu, Shi-Li Zhang & Zhi-Jun Qiu

Supplementary Information:

1. Extraction of the Schottky barrier height (SBH)

The output characteristics (I_d-V_d) of our multilayer MoS_2 FETs at different V_g are shown in Fig. S1a. Its linear and symmetric property indicates a small Schottky barrier height (SBH) formed at the MoS_2/metal interface. In order to quantitatively analyze the metal-semiconductor contact, the electron SBH, ϕ_{bn}, was obtained by performing temperature-dependent electrical measurements. According to the thermionic emission theory, I_d is related to ϕ_{bn} through the expression[S1]:

$$I_d = AA^*T^2 exp\left(-\frac{\Phi_{bn}}{kT}\right)\left[exp\left(\frac{qV_d}{kT}\right)-1\right]$$

where A is the area of the metal contact, A^* is the Richardson constant, T is the temperature, q is the electron charge and k is the Boltzmann constant. From the slope of the $\ln(I_d/T^2)$ versus 1/T plot, as shown in the Fig. S1b, ϕ_{bn} can be extracted. The ϕ_{bn} obtained with this approach is plotted in Fig. S1c as a function of V_g and its clear dependence on V_g reflects a gate-controlled metal-semiconductor barrier modulation. It decreases from 80 meV to near 0 eV as V_g is increased from -10 to 40 V, which is consistent with other reports[S2,S3].

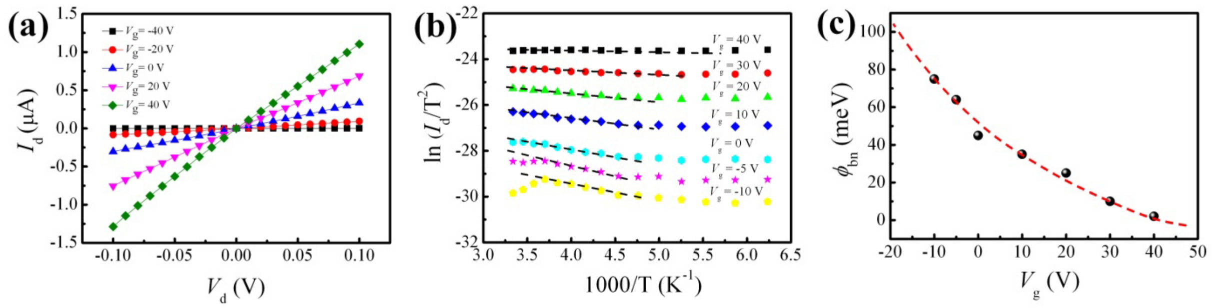

Figure S1. (a) Output characteristics of a representative transistor with a 10-nm-thick MoS_2 film. (b) Arrhenius plot $\ln(I_d/T^2)$ vs 1/T at different V_g. The dashed lines at high temperatures are exponential fittings to extract the electron SBH, ϕ_{bn}. (c) Extracted ϕ_{bn} as a function of V_g. The red dashed line serves as a guide to the eye.

[S1] Sze, S. M. *Physics of Semiconductor Devices*, 2nd ed.; John Wiley & Sons, Inc.: New York, 1981.

[S2] Das, S., Chen, H.-Y., Penumatcha, A. V. & Appenzeller, J. High performance multilayer MoS_2 transistors with scandium contacts. *Nano Lett.* **13,** 100-105 (2013).

[S3] Liu, H., Neal, A. T. & Ye, P. D. Channel length scaling of MoS_2 MOSFETs. *ACS Nano* **6,** 8563-8569 (2012).

2. Transfer characteristics of multilayer-MoS_2 FETs

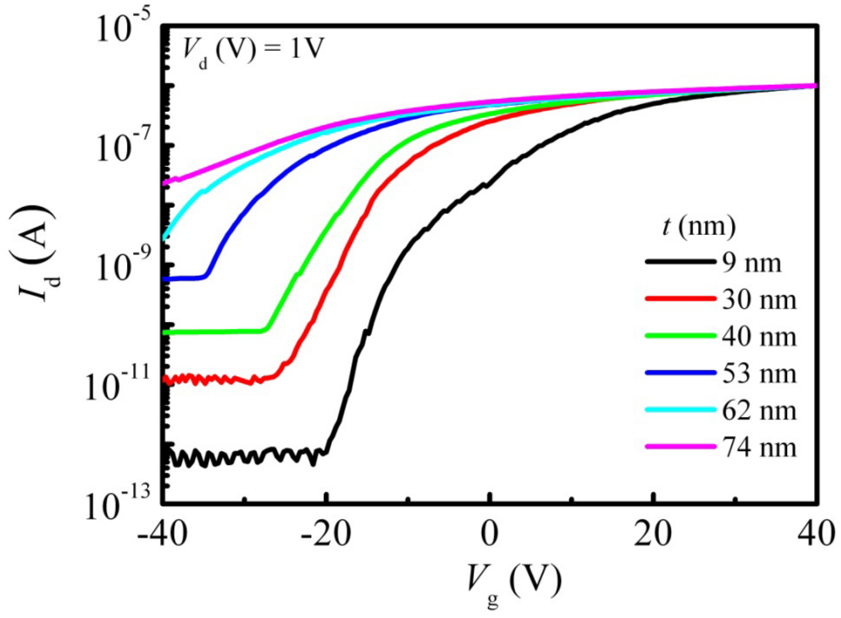

Figure S2. Transfer characteristics of multilayer-MoS_2 FETs with different MoS_2 film thickness.

3. Material parameters

Table S1: The material properties that were used in the TCAD simulation.

Material	Relative dielectric constant	Conduction band degeneracy	Electron effective mass	Hole effective mass	Electron mobility
MoS_2	11	6	$0.5m_e$	$1m_e$	30 cm^2/Vs

m_e is the mass of electron.
